# Suicidality and protective factors among sexual and gender minority youth and adults in Canada: a cross-sectional, population-based study

**DOI:** 10.1186/s12889-023-16285-4

**Published:** 2023-08-02

**Authors:** Li Liu, Brice Batomen, Nathaniel J. Pollock, Gisèle Contreras, Beth Jackson, Saiyi Pan, Wendy Thompson

**Affiliations:** 1 Public Health Agency of Canada, 785 Carling Avenue, Ottawa, ON K1S 5H4 Canada; 2 Dalla Lana School of Public Health, University of Toronto, Toronto, Canada; 3 School of Arctic and Subarctic Studies, Labrador Campus, Memorial University, Happy Valley-Goose Bay, Newfoundland and Labrador, Canada

**Keywords:** Suicidal ideation, Suicide attempt, LGBTQ2, Gay, Lesbian, Bisexual, Queer, Transgender, Social support, Health care access, COVID-19 pandemic

## Abstract

**Background:**

Sexual and gender minority populations experience elevated risks for suicidality. This study aimed to assess prevalence and disparities in non-fatal suicidality and potential protective factors related to social support and health care access among sexual and gender minority youth and adults and their heterosexual and cisgender counterparts in Canada. The second objective was to examine changes in the prevalence of suicidal ideation and protective factors during the COVID-19 pandemic.

**Methods:**

Pooled data from the 2015, 2016 and 2019 Canadian Community Health Surveys were used to estimate pre-pandemic prevalence of suicidal ideation, plans and attempts, and protective factors. The study also estimated changes in the prevalence of recent suicidal ideation and protective factors in fall 2020, compared with the same period pre-pandemic.

**Results:**

The prevalence of suicidality was higher among the sexual minority populations compared with the heterosexual population, and the prevalence was highest among the bisexual population, regardless of sex or age group. The pre-pandemic prevalence of recent suicidal ideation was 14.0% for the bisexual population, 5.2% for the gay/lesbian population, and 2.4% for the heterosexual population. The prevalence of lifetime suicide attempts was 16.6%, 8.6%, and 2.8% respectively. More than 40% of sexual minority populations aged 15–44 years had lifetime suicidal ideation; 64.3% and 36.5% of the gender minority population had lifetime suicidal ideation and suicide attempts. Sexual and gender minority populations had a lower prevalence of protective factors related to social support and health care access. The prevalence of recent suicidal ideation among sexual and gender minority populations increased in fall 2020, and they tended to experience longer wait times for immediate care needed.

**Conclusions:**

Sexual and gender minority populations had a higher prevalence of suicidality and less social support and health care access compared to the heterosexual and cisgender populations. The pandemic was associated with increased suicidal ideation and limited access to care for these groups. Public health interventions that target modifiable protective factors may help decrease suicidality and reduce health disparities.

## Highlights

• This study provides population-based pre-pandemic estimates of the prevalence of non-fatal suicidality (suicidal ideation, plans, and attempts) among sexual and gender minority populations in Canada using pooled data from the 2015, 2016, and 2019 Canadian Community Health Survey.

• Sexual and gender minority populations had a higher prevalence of suicidal ideation, plans, and attempts, compared with heterosexual and cisgender populations, regardless of sex or age group. Among sexual minority populations the bisexual population had the highest prevalence of suicidality.

• More than 40% of sexual minority populations aged 15 to 44 years had lifetime suicidal ideation; two thirds and one third of the gender minority population had lifetime suicidal ideation and suicide attempts, respectively.

• Sexual minority populations had a lower prevalence of social support and health care access; the presence of social support and health care access was related to lower prevalence of recent suicidal ideation.

• The onset of the COVID-19 pandemic was associated with increased recent suicidal ideation among the sexual and gender minority populations, especially for males, adolescents, and young adults.

## Background

Sexual and gender minority populations face unique stressors — homophobia and transphobia; violence; concealment of sexual identity; sexual orientation or gender identity conversion effects; and internalization of negative social attitudes [1–4]. Stigma-induced stress disrupts psychosocial wellbeing, a consequence of which may be a high prevalence of suicidality, including suicidal ideation, plans, and attempts [3–14]. A meta-analysis of 30 cross-sectional studies from high-income countries reported prevalence of lifetime suicide attempts among sexual minority adults ranging from 10% in population surveys to 20% in community surveys compared to 4% for heterosexual respondents [7]**.** Cross-sectional studies based on respondent-driven sampling in Ontario, the largest province in Canada, reported that one-third of transgender people had attempted suicide during their lifetime and 10% in the past year, compared with 3.7% and 0.6%, respectively, of the general population [8, 15, 16]. Similar patterns were observed in Europe and the United States (US) [9, 10, 14].

The prevalence of suicidality varies among sexual and gender minority populations, with elevated levels of suicidal ideation and suicide attempts among bisexual people, compared with those who are gay or lesbian [5, 17]. An analysis of combined data from the US and Canada revealed a bimodal age distribution in the prevalence of suicide attempts among sexual minority populations, with the first peak among 18 to 24 year-olds for both males and females, and the second among 30 to 35 year-olds for men, and 35 to 40 year-olds for women [18]. A 2022 Canadian study found that transgender 15 to 17 year-olds had five times the risk of suicidal ideation and 7.6 times the risk of suicide attempts, compared with cisgender (a person whose gender identity corresponds to the sex assigned at birth), heterosexual adolescents [19]. In the US, sexual minority youths were nearly 4 times more likely to contemplate suicide than their heterosexual counterparts [20]. Suicidal ideation and attempts were also associated with other psychosocial problems [21].

With the onset of the COVID-19 pandemic, suicidal ideation became more prevalent in many countries [22–28], including Canada [29, 30]. Sexual and gender minority populations were especially vulnerable to economic hardship [31], mental and emotional stress due to isolation, and decreased access to medical services [32], all of which were related to an increased risk of suicidality [33]. Three cycles of a cross-sectional Canadian survey conducted in 2020 and 2021 revealed that LGBTQ2 (lesbian, gay, bisexual, transgender, queer, and two-spirit) respondents were 1.7 times more likely than other respondents to have had suicidal thoughts or feelings in the past two weeks [29, 34]. The pandemic may have exacerbated symptoms of mental illness, especially among sexual and gender minority populations [35, 36].

Accurate estimates of suicidality among sexual and gender minority populations are necessary for public health surveillance and research [37]. A shortcoming of previous Canadian studies is that many were based on community surveys rather than population surveys, including several that used respondent-driven sampling methods [8, 15, 16]. Community surveys are subject to selection bias, as they over represent high-income sexual minorities and people with strong sexual and gender minority community attachment [7]. A recent population-based study used representative data from a national survey of children and youth, but by design was not able to examine prevalence among adults [19]. In addition, some Canadian pandemic studies lacked a pre-pandemic baseline [29], without which it is difficult to draw conclusions about temporal changes and the impact of the pandemic. Finally, modifiable factors that might reduce risks of suicidality, such as social support and health care access [8, 38, 39], have not been adequately examined in population-based studies of sexual and gender minority groups.

The objectives of this study were to: (1) estimate the prevalence of suicidal ideation, suicide plans, and suicide attempts among sexual and gender minority youth and adults in Canada before the pandemic, and compare it to heterosexual or cisgender counterparts; (2) estimate the prevalence of the factors related to social support and health care access and their correlates with suicidality; and (3) examine changes in the prevalence of recent suicidal ideation and associated factors among sexual and gender minority populations during the pandemic, compared with the pre-pandemic period.

## Methods

### Data source

The data were from the 2015, 2016, 2019, and 2020 Canadian Community Health Survey (CCHS) [40–43]. The 2017 and 2018 CCHS were not included because suicide-related questions were not asked in those years. The CCHS collects cross-sectional information on health outcomes, behaviours, and health care use from a nationally representative sample of the population aged 12 or older. The survey excludes full-time members of the Canadian Forces and people living on First Nations reserves/settlements, in foster homes, in two remote health regions in Quebec, and in institutions (healthcare institutions, prisons, religious institutions, etc.) [40–43]. The CCHS covers approximately 98% of the population aged 12 years or older in Canada [40–43].

For the population aged 18 years or older, the sample was selected from the area designed to serve the Labour Force Survey, which uses a probability sample that is based on a stratified multi-stage design. Respondents’ data are collected by a combination of computer-assisted personal and telephone interview software. The Canada Child Benefit frame is used to sample 12- to 17-year-olds; data from this frame are collected by telephone interview [40–43].

The CCHS response rates were 57.5% (2015), 61.3% (2016), 54.4% (2019), and 28.9% (2020). This study included respondents aged 15 years or older because younger respondents were not asked about sexual orientation or suicidality. The study includes only the respondents living in the 10 provinces but not in the territories, because the territories were sampled on a two-year basis; the data collected in 2019 offer only partial coverage and are not comparable with 2020 data.

### Measures

#### Suicidality

Three aspects of non-fatal suicidality were examined: suicidal ideation, suicide plans, and suicide attempts. Lifetime suicidal ideation was determined with the question: “Have you ever seriously contemplated suicide?” Among those who answered “yes,” recent ideation was determined by asking: “Has this happened in the past 12 months?” Lifetime and recent suicide plans were determined by the questions: “Have you ever made a plan to seriously attempt suicide” and “Has this happened in the past 12 months?” Lifetime and recent suicide attempts were determined by the questions: “Have you ever seriously attempted suicide” and “Has this happened in the past 12 months?” All of these questions produce prevalence estimates that are aligned with the Public Health Agency of Canada’s national Suicide Surveillance Indicator Framework [38].

#### Sexual orientation

In the 2015 and 2016 CCHS, sexual orientation was determined by asking: “Do you consider yourself to be…?” Response options were “heterosexual,” “homosexual, that is lesbian or gay,” and “bisexual.” The question in the 2019 and 2020 CCHS was: “What is your sexual orientation?” The response options were “heterosexual,” “gay or lesbian,” “bisexual or pansexual,” and “please specify.” All respondents who did not report heterosexual were coded as sexual minority. Those who did not choose a response option were coded “unknown.”

#### Gender identity

Gender identity was derived from questions about self-identified gender and sex assigned at birth. Respondents were asked “What is your gender?” (response options of “female,” “male” and “specify diverse”); the options for sex at birth were “female” and “male.” Respondents who did not specify gender “female” or “male” or whose gender did not match their sex at birth were classified as gender minority. Those who did not select a response option were coded “unknown.” Because gender identity was not asked in the 2015 and 2016 CCHS, only 2019 and 2020 data were used in the gender identity analyses.

#### Social support and health care access

To assess social support, six items from the CCHS were examined. Item (1) assessed community belonging [44]; items (2) to (6) have been used for the Social Provisions Scale (SPS-5), which measures reliable alliance, social integration, guidance, reassurance of worth, and attachment [39, 44].

1. **“**How would you describe your sense of belonging to your local community?”
2. “I have close relationships that provide me with a sense of emotional security and wellbeing.”
3. “There is someone I could talk to about important decisions in my life.”
4. “I have relationships where my competence and skill are recognized.”
5. “I feel part of a group of people who share my attitudes and beliefs.”
6. “There are people I can count on in an emergency.”

For item (1), sense of belonging to a community, the response options were “Very strong,” “Somewhat strong,” “Somewhat weak,” and “Very weak.” Those who responded “Very strong” or “Somewhat strong” were coded as having high community belonging [44].

For items (2) to (6), the response options were “Strongly agree,” “Agree,” “Disagree,” and “Strongly disagree.” Those who responded “Strongly agree” or “Agree” were coded as having high social support. In addition, the four responses from “Strongly disagree” to “Strongly agree” were scored as 1 to 4, and the mean social provisions score was computed. Respondents scoring 3 or more were identified as having a high level of social provisions [39, 44]. The mean score was not computed for respondents with missing data on any of the items (2) to (6).

Health care access was determined with six items. The first five items were based on the following questions:

1. “When you need immediate care for a minor health problem, how long do you usually have to wait before you can have an appointment?” The response options were: “on the same day,” “the next day,” “in 2 to 3 days,” “in 4 to 6 days,” “in 1 to 2 weeks,” “between 2 weeks and one month,” and “one month or more.” Responses were dichotomized as 0 to 3 days and more than 3 days.
2. “Do you have a regular health care provider?”
3. “During the past 12 months, was there ever a time when you felt that you needed health care, other than home care services, but you did not receive it?”
4. “Do you have insurance that covers all or part of the cost of your prescription medications?”
5. “Do you have insurance that covers all or part of your long-term care costs, including home care?”

The response options for items (2) to (5) were “yes” and “no.” The sixth item, on mental health related service needs met, was based on a derived variable in the CCHS. This variable is a summary classification of the respondent's overall perceived need for services for problems related to emotions, mental health or use of alcohol and drugs in the past 12 months. Respondents were grouped into one of four categories based on whether a need was reported (information, medication, counselling, other). Those with perceived needs that were all met were coded as “Yes”, while those with perceived needs that were only partially met or not met were coded as “No”.

#### Age group

The following age groups were used in the study: adolescent youth and emerging adults (15–24 years), [45] young to middle-aged adults (25–44 years), middle-aged adults (45–64 years), and older adults (65 years or older).

### Analysis

The analyses were conducted using SAS Enterprise Guide version 7.1 (SAS Institute, Cary, NC, USA). All estimates were adjusted with sampling weights provided by Statistics Canada, including adjustment for non-response to ensure that the estimates were representative of the population. The prevalence of suicidality and potential protective factors, with 95% confidence intervals, were estimated using the bootstrap technique with 1,000 bootstrap weights. Analyses of the prevalence of suicidality, stratified by sex at birth and age group, for sexual orientation were also conducted.

To examine recent suicidal ideation and potential protective factors before and during the pandemic, “pre-pandemic” CCHS data (2015, 2016 and 2019) were pooled and compared with results from the 2020 CCHS. Because of the pandemic, the 2020 CCHS did not collect data from April through August. Consequently, for pre-pandemic versus pandemic comparisons, the analyses were limited to data collected during the September-through-December periods in each year. The sexual and gender minority population groups were compared with the heterosexual, cisgender population. The protective factor analyses exclude having insurance that covers medications or long-term care costs because in 2020 these questions were asked only in selected provinces, not nationwide.

This study aggregated a de-identified dataset available through a data sharing agreement between the Public Health Agency of Canada and Statistics Canada. In accordance with the federal government’s Tri-Council Policy Statement: Ethical Conduct for Research Involving Humans, the use of this dataset did not require research ethics board approval.

## Results

In the combined 2015, 2016, and 2019 data, of the total 147,793 respondents who answered the sexual orientation questions, 1,931 (1.5%, 95% CI: 1.4–1.7) were gay or lesbian, and 2,407 (1.8%, 95% CI: 1.7–2.0) were bisexual or pansexual. Among 58,796 respondents in the 2019 CCHS, 96 (0.2%, 95% CI: 0.1–0.3) respondents were classified as gender minority. The distributions of sociodemographic characteristics in each CCHS cycle are presented in Table 1. Apart from an increase in the percentage of respondents aged 65 or older (from 18.8% in 2015 to 22.7% in 2020), distributions were similar across survey cycles. From September to December, there were 44,938 respondents in the combined 2015, 2016, and 2019 pre-pandemic data; it was 24,926 respondents for the same period during the pandemic in 2020 (Table 1).

**Table 1:** Population distribution by sociodemographic characteristics, 2015, 2016, 2019 and 2020 Canadian Community Health Survey

Sociodemographic characteristics	Distribution, % (95% CI)				
	**Pre-pandemic**				**During pandemic**
	**2015 (*N* = 46,478)**	**2016 (*N* = 50,550)**	**2019 (*N* = 58,796)**	**Combined 2015, 2016 and 2019 (Sept – Dec) (*N* = 44,938)**	**2020 (Sept – Dec) (*N* = 24,926)**
**Sex at birth**					
Female	50.7 (50.6, 50.8)	50.7 (50.7, 50.8)	50.6 (50.6, 50.7)	50.2 (49.6, 50.9)	51.1 (50.5, 51.6)
Male	49.3 (49.2, 49.4)	49.3 (49.2, 49.3)	49.4 (49.3, 49.4)	49.8 (49.1, 50.4)	48.9 (48.4, 49.5)
**Age group (years)**					
15–24	14.9 (14.4, 15.5)	14.1 (13.7, 14.5)	13.9 (13.4, 14.4)	14.1 (13.5, 14.8)	12.8 (12.0, 13.6)
25–44	32.3 (31.7, 33.0)	32.6 (32.0, 33.2)	33.1 (32.5, 33.7)	34.3 (33.5, 35.0)	31.6 (30.7, 32.6)
45–64	33.9 (33.5, 34.3)	34.0 (33.6, 34.4)	32.3 (31.9, 32.6)	33.0 (32.4, 33.7)	32.8 (32.1, 33.5)
65 +	18.8 (18.8, 18.8)	19.3 (19.3, 19.3)	20.7 (20.7, 20.8)	18.6 (18.1, 19.1)	22.7 (22.4, 23.1)
**Sexual orientation**					
Heterosexual	89.3 (88.7, 89.8)	90.2 (89.7, 90.8)	91.1 (90.7, 91.6)	88.7 (88.1, 89.4)	91.2 (90.6, 91.8)
Gay and lesbian	1.4 (1.2, 1.6)	1.4 (1.2, 1.5)	1.6 (1.4, 1.7)	1.6 (1.4, 1.8)	1.7 (1.4, 2.0)
Bisexual and pansexual	1.5 (1.3, 1.7)	1.6 (1.4, 1.8)	2.0 (1.8, 2.2)	1.8 (1.6, 2.0)	1.9 (1.6, 2.2)
Other	N/A	N/A	0.2 (0.1, 0.3)	0.1 (0.1, 0.1)	0.3 (0.2, 0.5)
Unknown	7.8 (7.3, 8.3)	6.8 (6.3, 7.3)	5.1 (4.7, 5.5)	7.8 (7.1, 8.4)	4.9 (4.4, 5.3)
**Gender identity**^**a**^					
Gender minority	N/A	N/A	0.20 (0.13, 0.26)	0.21 (0.12, 0.30)^E^	0.34 (0.18, 0.50)^E^
Cisgender	N/A	N/A	99.8 (99.7, 99.9)	99.8 (99.7, 99.9)	99.6 (99.5, 99.8)
Unknown	N/A	N/A	0.02 (0.00, 0.03)	0.01 (0.00, 0.01)	0.04 (0.00, 0.10)

*Abbreviation*: *CI* Confidence interval, *N/A* Data not available

^a^ Data for gender identity not available in 2015 and 2016

^E^ Interpret with caution due to high sampling variability

### Pre-pandemic suicidal ideation, suicide plans, and suicide attempts

The prevalence of suicidal ideation, suicide plans, and suicide attempts during the three-year pre-pandemic period by sexual orientation are reported in Table 2. The prevalence of recent suicidal ideation was 2.4% (95% CI: 2.3–2.5) among the heterosexual population, 5.2% (95% CI: 4.1–6.5) among the gay and lesbian population, and 14.0% (95% CI: 11.8–16.5) among the bisexual and pansexual population; the corresponding percentage reporting lifetime suicide attempts were 2.8% (95% CI: 2.7–3.0), 8.6% (95% CI: 6.8–10.8), and 16.6% (95% CI: 14.2–19.2). The same pattern emerged for the other suicide-related outcomes, both recent and lifetime, with the heterosexual population having the lowest prevalence, and the bisexual and pansexual populations, the highest. This pattern persisted in sex-stratified analyses. Among both the sexual minority and heterosexual populations, females and younger people tended to have a higher prevalence of each outcome. More than 40% of sexual minority populations aged 15–44 years had suicidal ideation during their life-time.

**Table 2:** Prevalence of suicidal ideation, plans, and attempts, by sexual orientation, 2015, 2016 and 2019 combined, Canada

Suicide outcome	Prevalence, % (95% CI)			
	**Heterosexual**	**Sexual Minority**		
		**Gay and lesbian**	**Bisexual and pansexual**	**Sexual minority overall**
**Suicidal ideation (past 12 months)**				
**Overall**	2.4 (2.3, 2.5)	5.2 (4.1, 6.5)	14.0 (11.8, 16.5)	10.1 (8.8, 11.5)
**Sex at birth**				
Female	2.5 (2.4, 2.7)	6.8 (4.7, 9.4)^E^	15.8 (13.0, 19.0)	13.0 (10.9, 15.3)
Male	2.2 (2.0, 2.4)	4.3 (3.0, 5.8)^E^	10.2 (7.1, 14.1)^E^	6.6 (5.2, 8.4)
**Age group (years)**				
15–24	5.0 (4.5, 5.7)	─^F^	─^F^	18.8 (15.5, 22.3)
25–44	2.5 (2.3, 2.7)	─^F^	─^F^	9.3 (7.2, 11.8)
45–64	1.9 (1.7, 2.1)	─^F^	─^F^	3.9 (2.7, 5.5)^E^
65 or older	1.1 (0.9, 1.3)	─^F^	─^F^	─^F^
**Suicidal ideation (lifetime)**				
**Overall**	11.5 (11.2, 11.8)	25.5 (22.3, 28.8)	46.7 (43.4, 50.0)	37.3 (34.9, 39.7)
**Sex at birth**				
Female	12.5 (12.1, 12.9)	28.3 (23.8, 33.2)	53.3 (49.3, 57.3)	45.6 (42.4, 48.8)
Male	10.4 (10.1, 10.8)	23.8 (19.5, 28.5)	32.8 (27.8, 38.2)	27.3 (23.9, 30.9)
**Age group (years)**				
15–24	12.8 (12.0, 13.7)	─^F^	─^F^	43.8 (39.2, 48.6)
25–44	12.7 (12.2, 13.2)	─^F^	─^F^	40.0 (36.3, 43.7)
45–64	12.0 (11.5, 12.5)	─^F^	─^F^	31.9 (27.0, 37.1)
65 or older	7.3 (6.9, 7.6)	─^F^	─^F^	14.7 (11.2, 18.9)
**Suicide plans (past 12 months)**				
**Overall**	0.6 (0.6, 0.7)	1.8 (1.3, 2.5)^E^	6.7 (5.1, 8.7)	4.5 (3.5, 5.6)
**Sex at birth**				
Female	0.8 (0.7, 0.9)	2.6 (1.6, 4.1)^E^	7.6 (5.5, 10.2)^E^	6.0 (4.5, 7.8)
Male	0.5 (0.4, 0.6)	1.3 (0.7, 2.2)^E^	4.8 (2.4, 8.5)^E^	2.6 (1.6, 4.1)^E^
**Age group (years)**				
15–24	1.5 (1.2, 1.8)	─^F^	─^F^	8.3 (6.2, 10.8)
25–44	0.6 (0.5, 0.8)	─^F^	─^F^	4.3 (2.7, 6.5)^E^
45–64	0.5 (0.4, 0.6)	─^F^	─^F^	1.6 (0.9, 2.5)^E^
65 or older	0.3 (0.2, 0.3)	─^F^	─^F^	─^F^
**Suicide plans (lifetime)**				
**Overall**	3.8 (3.7, 4.0)	12.1 (10.1, 14.3)	24.7 (21.8, 27.8)	19.1 (17.3, 21.0)
**Sex at birth**				
Female	4.4 (4.2, 4.7)	14.8 (11.4, 18.7)	28.4 (24.6, 32.3)	24.2 (21.5, 27.1)
Male	3.2 (3.0, 3.5)	10.5 (8.0, 13.4)	17.2 (13.0, 22.3)	13.0 (10.7, 15.6)
**Age group (years)**				
15–24	4.8 (4.3, 5.4)	─^F^	─^F^	25.2 (20.9, 29.7)
25–44	4.5 (4.2, 4.8)	─^F^	─^F^	19.6 (16.8, 22.6)
45–64	3.8 (3.5, 4.0)	─^F^	─^F^	15.0 (11.8, 18.7)
65 or older	2.1 (1.9, 2.3)	─^F^	─^F^	6.4 (4.1, 9.5)^E^
**Suicide attempts (past 12 months)**				
**Overall**	0.3 (0.3, 0.4)	0.6 (0.3, 1.1)^E^	4.1 (2.6, 6.1)^E^	2.6 (1.7, 3.6)^E^
**Sex at birth**				
Female	0.4 (0.3, 0.5)	─^F^	4.2 (2.3, 6.8)^E^	3.2 (1.9, 5.0)^E^
Male	0.2 (0.2, 0.3)	─^F^	3.9 (1.8, 7.4)^E^	1.8 (0.9, 3.1)^E^
**Age group, years**				
15–24	1.0 (0.7, 1.2)	─^F^	─^F^	4.2 (2.6, 6.4)^E^
25–44	0.3 (0.2, 0.3)	─^F^	─^F^	2.8 (1.3, 5.2)^E^
45–64	0.2 (0.2, 0.3)	─^F^	─^F^	0.9 (0.4, 1.6)^E^
65 or older	0.1 (0.1, 0.2)^E^	─^F^	─^F^	─^F^
**Suicide attempts (lifetime)**				
**Overall**	2.8 (2.7, 3.0)	8.6 (6.8, 10.8)	16.6 (14.2, 19.2)	13.1 (11.6, 14.8)
**Sex at birth**				
Female	3.5 (3.3, 3.7)	8.6 (6.2, 11.6)^E^	17.7 (14.8, 20.9)	15.1 (12.9, 17.5)
Male	2.1 (2.0, 2.3)	8.7 (6.2, 11.7)^E^	14.1 (10.2, 18.9)^E^	10.7 (8.5, 13.3)
**Age group (years)**				
15–24	3.4 (3.0, 3.9)	─^F^	─^F^	14.7 (11.8, 18.0)
25–44	3.2 (2.9, 3.5)	─^F^	─^F^	14.6 (11.9, 17.6)
45–64	2.9 (2.7, 3.2)	─^F^	─^F^	11.3 (8.6, 14.6)
65 or older	1.5 (1.4, 1.7)	─^F^	─^F^	4.1 (2.4, 6.5)^E^

Among heterosexual and gender minority samples, fewer than 0.3% had missing data on suicide outcomes and were excluded from analyses

Data source: Canadian Community Health Survey

*Abbreviation*: *CI* Confidence interval

^E^ Interpret with caution due to high sampling variability

^F^ Data not reliable due to high sampling variability

The gender minority population had a higher prevalence of all outcomes, compared with the cisgender population (Table 3). Nearly two-thirds of the gender minority population had suicidal ideation and one third had suicide attempts during their lifetime. The gender minority population had 10 times and 5 times the prevalence of recent and lifetime suicidal ideation, respectively, and 12 times the prevalence of lifetime suicide attempts.

**Table 3:** Prevalence of suicidal ideation, plans, and attempts, by gender identity, 2019, Canada

Suicide outcome	Prevalence, % (95% CI)	
	**Cisgender**	**Gender minority**
**Suicidal ideation (past 12 months)**	2.8 (2.6, 3.0)	28.2 (12.1, 49.8)^E^
**Suicidal ideation (lifetime)**	12.5 (12.0, 13.0)	64.3 (45.4, 80.4)
**Suicide plans (past 12 months)**	0.8 (0.7, 0.9)	─^F^
**Suicide plans (lifetime)**	4.4 (4.1, 4.7)	23.4 (11.8, 38.9)^E^
**Suicide attempts (past 12 months)**	0.3 (0.3, 0.4)	─^F^
**Suicide attempts (lifetime)**	3.0 (2.8, 3.3)	36.5 (19.6, 56.3)^E^

Among cisgender and gender minority samples, 3% had missing data on suicide outcomes and were excluded from analyses

Data source: Canadian Community Health Survey

*Abbreviation*: *CI* Confidence interval

^E^ Interpret with caution due to high sampling variability

^F^ Data not reliable due to high sampling variability

### Pre-pandemic social support and health care access

Sexual and gender minority populations had relatively low levels of social support and health care access (Table 4). However, among these groups, most dimensions of social support and health care access were associated with decreased suicidal ideation. People who had a high level of social provisions had lower prevalence of reporting suicidal ideation—(8.2%, 95% CI: 6.4–9.9)—in comparison with people whose level of social provisions was not high (17.8%, 95% CI: 14.2–21.5). Similarly, receiving health care when it was needed was associated with much lower prevalence of suicidal ideation: 9.2% (95% CI: 7.1–11.3) vs. 24.4% (15.0–33.9).

**Table 4:** Prevalence of protective factors and associations with suicidal ideation in past 12 months among sexual and gender minority populations, 2015, 2016, and 2019 combined, Canada

Protective factors	Prevalence of protective factor, % (95% CI)		Prevalence of suicidal ideation among sexual and gender minority populations % (95% CI)	
	**Heterosexual, cisgender**	**Sexual and gender minority**^**a**^	**Yes on protective factor**	**No on protective factor**
**Social support**				
Strong community belonging	68.5 (68.1, 68.9)	57.9 (55.3, 60.4)	8.8 (6.9, 10.8)	12.4 (10.3, 14.5)
High level of social provisions^b^	74.9 (74.4, 75.3)	72.6 (70.1, 75.0)	8.2 (6.4, 9.9)	17.8 (14.2, 21.5)
Having relationships providing emotional security	96.6 (96.4, 96.8)	94.0 (92.8, 95.2)	9.2 (7.7, 10.8)	29.6 (20.3, 38.9)^E^
Having someone to talk about important decisions	97.1 (96.9, 97.2)	95.6 (94.4, 96.8)	9.8 (8.2, 11.4)	24.7 (14.3, 35.0)^E^
Competence recognized	96.5 (96.3, 96.7)	94.5 (93.4, 95.6)	9.4 (7.9, 11.0)	28.5 (19.9, 37.1)^E^
Having relationships to share attitudes and beliefs	93.5 (93.2, 93.7)	89.5 (87.9, 91.1)	8.7 (7.2, 10.3)	26.0 (19.9, 32.0)
Having someone to count on in emergency	98.4 (98.3, 98.6)	97.1 (96.2, 98.0)	9.9 (8.4, 11.5)	28.0 (14.9, 41.1)^E^
**Access to care**				
Wait time within 3 days for immediate care needs	63.8 (63.4, 64.3)	56.8 (54.0, 59.7)	8.3 (6.5, 10.2)	13.5 (10.8, 16.3)
Having regular health care provider	84.1 (83.7, 84.4)	78.9 (76.9, 80.9)	10.5 (9.0, 12.1)	9.4 (5.8, 12.9)^E^
Health care received when needed	95.9 (95.7, 96.2)	89.8 (87.8, 91.7)	9.2 (7.1, 11.3)	24.4 (15.0, 33.9)^E^
Having medication insurance	80.6 (80.2, 81.0)	78.1 (75.9, 80.4)	9.7 (8.1, 11.2)	12.1 (8.6, 15.7)
Having long-term care insurance	49.2 (48.7, 49.7)	45.7 (43.0, 48.5)	7.5 (5.5, 9.5)	11.4 (9.0, 13.7)
All perceived mental health needs met^c^	55.4 (53.6, 57.1)	48.9 (43.5, 54.3)	20.0 (14.0, 26.0)	23.0 (16.6, 29.3)

Among heterosexual, cisgender and sexual and gender minority samples, fewer than 2% had missing data for most protective factors, except waiting time (5%), mental health needs (5%) and long-term care insurance (16%), and were excluded from the analyses

Data source: Canadian Community Health Survey

*Abbreviation*: *CI* Confidence interval

^a^ Data for gender minority not available in 2015 and 2016

^b^ Data for social provisions not available in 2015

^c^ Data for mental health needs met not available in 2015 and 2016

^E^ Interpret with caution due to high sampling variability

### Recent suicidal ideation and protective factors before and during the pandemic

The prevalence of recent suicidal ideation among sexual and gender minority populations versus the heterosexual, cisgender population before and during the pandemic are reported in Table 5. A comparison of the prevalence of recent suicidal ideation in September-through-December period in 2020 with pooled data for the same four months in 2015, 2016 and 2019 show an increase from 8.5% (95% CI: 6.4–11.1) to 13.3% (95% CI: 9.5–17.9) among the sexual and gender minority populations. By contrast, prevalence decreased slightly among their heterosexual, cisgender counterparts. The increase among sexual and gender minority populations was higher for males and 15- to 24-year-olds.

**Table 5:** Prevalence of recent suicidal ideation before COVID-19 pandemic (2015, 2016 and 2019 combined, September through December) versus and during pandemic (2020, September through December), Canada

	**Populations**^**a**^	**Prevalence, % (95% CI)**		
		**Combined 2015, 2016 and 2019**^**a**^	**2020**	**Difference (2020 - combined)**
**Overall**				
All	Sexual and gender minority	8.5 (6.4, 11.1)	13.3 (9.5, 17.9)^E^	4.8 (0.2, 9.3)^E^
	Heterosexual, cisgender	2.4 (2.2, 2.7)	2.0 (1.7, 2.4)	-0.4 (-0.8, 0.0)
**Sex**				
Female	Sexual and gender minority	11.5 (8.3, 15.3)^E^	12.8 (8.5, 18.1)^E^	1.3 (-4.3, 6.9)^E^
	Heterosexual, cisgender	2.5 (2.2, 2.8)	2.2 (1.8, 2.8)	-0.2 (-0.8, 0.4)
Male	Sexual and gender minority	4.9 (2.8, 8.0)^E^	14.0 (7.6, 22.8)^E^	9.0 (1.4, 16.7)^E^
	Heterosexual, cisgender	2.4 (2.0, 2.8)	1.8 (1.3, 2.3)	-0.6 (-1.2, 0.0)
**Age group (years)**				
15–24	Sexual and gender minority	17.3 (11.6, 24.3)^E^	25.4 (15.8, 37.3)^E^	8.2 (-3.7, 20.1)^E^
	Heterosexual, cisgender	5.4 (4.3, 6.7)	3.6 (2.4, 5.3)^E^	-1.8 (-3.5, 0.0) ^E^
25 or older	Sexual and gender minority	5.5 (3.6, 7.8)^E^	7.7 (5.0, 11.3)^E^	2.3 (-1.3, 5.9)^E^
	Heterosexual, cisgender	1.9 (1.7, 2.2)	1.8 (1.4, 2.2)	-0.2 (-0.6, 0.3)

Among sexual and gender minority and heterosexual, cisgender samples, fewer than 0.3% had missing suicide outcome data and were excluded from analyses

*Abbreviation*: *CI* Confidence intervals

^a^ Data for gender minority data not available in 2015 and 2016

^E^ Interpret with caution due to high sampling variability

When comparing the fall of 2020 with the same four months in previous years, the prevalence of social support and health care access did not decrease (except wait time for immediate care), and disparities between the sexual and gender minority populations and the heterosexual, cisgender population did not widen (Table 6). Fewer people reported wait times within 3 days for immediate care needs, regardless of their sexual orientation or gender identity, but the percentage-point drop was greater among sexual and gender minority populations, 8.8 (95% CI: -16.8, -0.7) percentage points reduction, compared to -3.1 (95% CI: -4.6–-1.6) for the heterosexual, cisgender population.

**Table 6:** Prevalence of protective factors before COVID-19 pandemic (2015, 2016 and 2019 combined, September through December) versus during pandemic (2020, September through December), Canada

Protective factors	Populations^a^	Prevalence, % (95% CI)		
		**Combined 2015, 2016 and 2019**	**2020**	**Difference**
**Social support**				
Strong community belonging	Sexual and gender minority	55.0 (50.1, 59.8)	61.2 (55.8, 66.4)	6.2 (-0.8, 13.3)
	Heterosexual, cisgender	68.3 (67.5, 69.1)	70.0 (68.9, 71.1)	1.7 (0.4, 3.0)
High level of social provisions^**b**^	Sexual and gender minority	71.3 (66.5, 75.7)	72.6 (64.2, 80.0)	1.3 (-7.8, 10.5)
	Heterosexual, cisgender	74.3 (73.5, 75.2)	75.7 (74.1, 77.2)	1.4 (-0.4, 3.1)
Having relationships providing emotional security	Sexual and gender minority	93.6 (91.0, 95.6)	95.0 (90.9, 97.6)	1.5 (-2.3, 5.2)
	Heterosexual, cisgender	96.6 (96.2, 96.9)	96.8 (96.1, 97.4)	0.2 (-0.5, 0.9)
Having someone to talk about important decisions	Sexual and gender minority	95.6 (93.4, 97.3)	95.6 (91.4, 98.1)	0.0 (-3.5, 3.5)
	Heterosexual, cisgender	97.2 (96.9, 97.5)	97.3 (96.6, 97.9)	0.0 (-0.7, 0.7)
Competence recognized	Sexual and gender minority	94.7 (92.7, 96.3)	94.0 (89.3, 97.1)	-0.7 (-4.8, 3.4)
	Heterosexual, cisgender	96.3 (96.0, 96.7)	96.4 (95.5, 97.1)	0.0 (-0.8, 0.9)
Having relationships to share attitudes and belief	Sexual and gender minority	89.2 (86.1, 91.8)	87.5 (81.1, 92.4)	-1.6 (-7.7, 4.4)
	Heterosexual, cisgender	93.4 (93.0, 93.9)	94.3 (93.3, 95.1)	0.8 (-0.2, 1.9)
Have someone to count on in emergency	Sexual and gender minority	97.6 (96.1, 98.7)	96.7 (93.8, 98.5)	-0.9 (-3.4, 1.5)
	Heterosexual, cisgender	98.4 (98.1, 98.6)	98.3 (97.7, 98.8)	0.0 (-0.6, 0.5)
**Access to care**				
Wait time within 3 days for immediate care needs	Sexual and gender minority	54.4 (49.2, 59.7)	45.7 (39.5, 51.9)	-8.8 (-16.8, -0.7)
	Heterosexual, cisgender	63.1 (62.2, 64.0)	60.0 (58.8, 61.2)	-3.1 (-4.6, -1.6)
Having regular health care provider	Sexual and gender minority	76.2 (71.6, 80.4)	78.8 (72.8, 84.0)	2.7 (-4.3, 9.6)
	Heterosexual, cisgender	83.8 (83.1, 84.5)	86.5 (85.7, 87.3)	2.8 (1.7, 3.8)
Health care received when needed	Sexual and gender minority	89.4 (85.1, 92.8)	91.3 (87.6, 94.2)	1.9 (-2.9, 6.7)
	Heterosexual, cisgender	95.9 (95.4, 96.4)	94.4 (93.8, 95.0)	-1.5 (-2.2, -0.7)
All perceived mental health needs met^c^	Sexual and gender minority	43.1 (34.2, 52.4)	52.4 (44.3, 60.4)	-9.3 (-21.0, 2.3)
	Heterosexual, cisgender	54.1 (50.8, 57.3)	55.8 (52.8, 58.8)	-1.7 (-6.2, 2.7)

Among sexual and gender minority and heterosexual, cisgender samples, fewer than 2% had missing data for protective factors, except for waiting time and (5%) and mental health needs (5%), and were excluded from the analyses

*Abbreviation*: *CI* Confidence intervals

^a^ Gender minority data not available in 2015 and 2016

^b^ Social provisions data not available in 2015

^c^ Data for mental health needs met not available in 2015 and 2016

## Discussion

This study provides population-based estimates of the prevalence of suicidality among sexual and gender minority populations in Canada. The prevalence of all suicide-related outcomes was higher among the sexual and gender minority populations compared with the heterosexual and cisgender peers and differences were observed in all sex and age groups. The bisexual population had the highest prevalence of each outcome. Sexual and gender minority populations were less likely than the heterosexual, cisgender population to report social support and health care access, factors that were associated with reduced suicidal ideation. During the COVID-19 pandemic, more sexual and gender minority populations experienced suicidal ideation, especially for males, adolescents, and young adults. To our knowledge, this is the first Canadian study using population survey data to study suicidal behaviors and protective factors on social support and access to care among sexual and gender minority populations at a national level covered all age groups of the population. Moreover, this is also the first Canadian study that provides timely information to evaluate the impacts of the pandemic on this specific voluntary population group.

Our results are consistent with previous systematic reviews [5, 7, 17]. A meta-analysis of population-based surveys of lifetime suicide attempts among sexual minorities reported a prevalence of 11% (95% CI: 8% to 15%) [7], similar to the 13.1% (95% CI: 11.6% to 14.8%) observed in the present analysis, and consistent with our results, reported a higher prevalence among the bisexual population. Another systematic review found the lifetime risk of suicide attempts to be especially high among gay and bisexual males [5]. The inclusion of non-population-based studies in the review may explain why results differed from our finding of a higher prevalence of suicidality among females for sexual minority populations. The higher prevalence of suicidality among bisexual women is worth noting, and suggests that intersecting systems of sexism and biphobia may exacerbate stressors on bisexual women [46].

Bisexual people have been found to have significantly poorer mental health and unique life experiences that distinguish them from other sexual minority groups [47, 48]. These differences may be related to the higher prevalence of suicidality observed among this group, compared with gay, lesbian or heterosexual populations. Studies have reported that bisexual people experience barriers to mental health and psychosocial support, and that services tailored to the needs of this population are limited [47, 48].

The higher prevalence of suicidality among sexual and gender minority adolescents and young adults observed in our analysis is similar to research indicating that recent suicide attempts peak at ages 18 to 20 [18]. A Canadian study of 15- to 17-year-olds found that 4.6% of heterosexual youth and 14.3% of bisexual youth (who accounted for about 90% of sexual minority youth) reported a suicide attempt during their lifetime; our results for 15- to 24-year-olds were similar—3.4% for those who were heterosexual and 14.7% for those who were sexual minorities [19]. However, the prevalence of recent suicidal ideation reported in the earlier study exceeded our estimates (10.4% versus 5.0% for heterosexual youth, and 28.8% for bisexual versus 18.8% for sexual minority youth) [19]. The lower prevalence in our results may partially reflect the differences in age ranges, as a higher prevalence of suicidal ideation at younger ages was found. Sexual minority youth may be particularly likely to experience difficulties related to emotional well-being, self-esteem, and family and school connectedness, all of which are related to mental health (including suicidal ideation) [49].

Based on a Canadian survey conducted in September 2020, the prevalence of recent suicidal ideation among the sexual and gender minority populations was 23% in September 2020 [34]; a much lower prevalence—12% was observed. The definition used in that survey to identify recent suicidal ideation (that is, any suicidal thoughts or feelings in the previous two weeks) may, in part, account for the discrepancy. That survey also found that cisgender men were more likely than cisgender women to report suicidal ideation [29].

In our analysis, the gender minority population accounted for 0.34% (95% CI: 0.18, 0.50) of the total population in 2020 (September to December). This figure was similar to the results of the 2021 Census—0.33% of the population aged 15 or older identified as trans or non-binary [50]. Similarly, a 2018 national survey estimated that trans/non-binary people made up 0.24% of the population aged 15 or older [51, 52]; our estimate for the gender minority population in 2019 was 0.21% (95% CI: 0.12, 0.30).

This study found that social support and access to health care were associated with lower prevalence of suicidal ideation among sexual minority populations in comparison to heterosexuals. These results align with research reporting that high levels of social support and changes in personal identification documents to reflect appropriate gender designation were associated with reductions in suicide attempts among transgender populations [8, 15].

These findings support the hypothesis that sexual and gender minority populations were at heightened risk of experiencing pandemic-related suicidal outcomes [33]. By contrast, for the heterosexual, cisgender population, the pandemic was not associated with an increased prevalence of recent suicidal ideation, but rather, a slight decrease. The relatively greater increase among 15- to 24-year-olds supports the hypothesis that young people were particularly vulnerable during the pandemic. This may be a consequence of fewer opportunities for peers to observe distress, greater substance use, grief due to family illness, death or economic loss, and lack of access to social support [53, 54].

### Limitations

This study examined suicide-related outcomes based on the pooled data from multiple cycles of the CCHS, one of the largest national representative health survey in Canada. However, several factors could bias the estimates presented in this study. The CCHS excludes only about 3% of the population aged 12 or older, but some excluded groups—notably, residents of remote regions, First Nation reserves, and institutions [43]—have a relatively higher prevalence of suicidality [29, 55–58]. As well, our analyses were based only on data from the 10 provinces; the territories were not included.

Potential misclassification and underreporting of sexual and gender minority and suicidality due to stigma are major limitations [7, 59]. In addition, some studies suggest that self-reported lifetime suicidal behaviours are inconsistent over time [60]. Although underreporting and errors in self-reported, suicidal outcomes can be thought to be similar among sexual and gender minority population and the general population, the misclassification of suicidality in each group is probably non-differential [61]. Some studies have found that sexual minorities are at greater risk of suicide attempts before coming out [62, 63]. The bias introduced by this misclassification could go in either direction [64].

Because the CCHS is cross-sectional, survival bias is possible; that is, only people who have survived suicide experiences can respond to the questionnaire. Therefore, the prevalence of suicidality (mostly attempts) is likely underestimated [7, 8]**.** As well, the extent of underestimation may not be the same for sexual and gender minority populations and the general population. Pre-existing mental health conditions have been associated with suicidal behaviours [34, 65], but the cross-sectional data do not permit adjustment for psychosocial well-being, which can be both a confounder and a mediator in the relationship between sexual orientation/gender identity and suicidality.

The potential protective factors evaluated in this analysis were proxies for complex constructs. Scales that have been developed for social support and health care access [66, 67] were not available in the CCHS. In addition, measures that quantify access to mental health care are lacking [68]. Owing to the pandemic, the response rate to the 2020 CCHS was low. Despite sample weights designed to take account of non-response, its impact is not completely known. In addition, although the proportion of missing data was very low (< 5%, with the exception of some protective factors), the likelihood of missing data may be related to respondents’ sexual orientation and gender identity, therefore introducing a selection bias [69].

## Conclusions

Sexual and gender minority populations had higher prevalence of suicidality and lower prevalence of social support and access to care, compared with the heterosexual and cisgender population. The prevalence of suicidality was higher among females, younger people, and the bisexual population. The COVID-19 pandemic was associated with increased suicidal ideation and wait time for immediate care need among sexual and gender minority populations.

Our findings reveal a need for public health policies and interventions designed to provide social support and address health care access issues, and thereby, help reduce suicidality among sexual and gender minority populations. This could include focusing on upstream determinants of health such as supportive public policies [2, 70] and anti-discrimination efforts.

## Data Availability

The datasets analyzed during the current study are available in the Statistics Canada Research Data Centres with data record number 3226. https://www.statcan.gc.ca/en/microdata/data-centres/data.

## Abbreviations

CCHS: Canadian Community Health Survey
CI: Confidence interval
LGBTQ2: Lesbian, gay, bisexual, transgender, queer, and two-spirit
